# The association between wearable device use and exercise adherence: a chain mediation model of exercise self-efficacy and positive experiences

**DOI:** 10.3389/fpsyg.2026.1943574

**Published:** 2026-10-29

**Authors:** Zitong Qin, Ying Guo, Xinlong Liu

**Affiliations:** 1 Faculty of Education, Universiti Malaya, Kuala Lumpur, Malaysia; 2 Capital Institute of Physical Education and Sports, Beijing, China; 3 Tianjin University, Tianjin, China

**Keywords:** chain mediation model, exercise persistence, exercise self-efficacy, positive experiences, structural equation modeling, wearable device use

## Abstract

**Objective:**

To examine the association between wearable device use and exercise adherence and to investigate the chain-mediated roles of exercise self-efficacy and positive experiences in this relationship.

**Methods:**

A questionnaire survey was administered to individuals who used fitness apps or smart wearable devices (*N* = 367). Structural equation modeling (SEM) was conducted to examine the hypothesized associations and mediation pathways. The Bootstrap method with 5,000 resamples was applied to assess indirect effects.

**Results:**

Wearable device use showed a statistically significant total association with exercise adherence (*β* = 0.430, *p* < 0.001); after the two proposed mediators were included, the direct association was *β* = 0.197 (*p* < 0.001). Exercise self-efficacy and positive experiences each showed significant independent indirect associations, and the sequential (chain) indirect pathway involving both variables was also statistically significant (indirect association = 0.059, 95% CI [0.035, 0.086]). The indirect associations statistically accounted for 54.0% of the total association.

**Conclusion:**

Among physically active users of digital fitness technologies, wearable device use was positively associated with exercise adherence, and this association was partially accounted for by a chain mediation pathway involving exercise self-efficacy and positive experiences. The findings are consistent with, but do not confirm, the proposed directional model; they identify psychological factors associated with exercise adherence among wearable device users and may inform future research on technology-assisted approaches for supporting sustained exercise behaviour.

## 1 Introduction

Wearable health devices (e.g., smartwatches and fitness trackers) have rapidly gained popularity worldwide, with their market size and user adoption showing sustained growth (Piwek et al., 2016; Brickwood et al., 2019). Equipped with functions such as real-time data tracking, goal setting and feedback, social interaction, and personalized recommendations, wearable devices are associated with changes in sedentary behaviour and physical activity levels (Warburton et al., 2006; Ringeval et al., 2020). Systematic reviews and meta-analyses indicate that consumer-based activity trackers are associated with modest increases in physical activity participation (Brickwood et al., 2019), although effects on longer-term behavioural outcomes remain heterogeneous, and digital behaviour-change interventions have been criticised for weak maintenance effects (Vandelanotte et al., 2016). However, research has also identified an “engagement–adherence paradox”: although many users initially intend to increase their physical activity after adopting wearable devices, a substantial proportion abandon the device or discontinue regular exercise within 6 months (Lazar et al., 2015; Fritz et al., 2014). This phenomenon suggests that the association between wearable device use and exercise adherence may not be straightforward and may involve complex psychological pathways.

Exercise adherence refers to an individual’s capacity and tendency to maintain regular exercise behaviour over an extended period and represents an important research topic in exercise psychology (Dishman, 1994; Rhodes and Rebar, 2018). Previous studies have identified significant associations between various psychological factors and exercise adherence, among which exercise self-efficacy and positive exercise experiences have been considered particularly important (Bandura, 1997; McAuley et al., 2011; Ekkekakis et al., 2021). Self-efficacy theory suggests that individuals’ beliefs regarding their ability to perform specific exercise tasks are closely associated with their persistence and effort when encountering challenges (Bandura, 1997; Luszczynska and Schwarzer, 2005). Meanwhile, positive emotional experiences during exercise may be associated with subsequent exercise intentions through affective memory processes, reflecting a potential reciprocal pattern between exercise participation and affective experiences (Ekkekakis, 2017; Ekkekakis et al., 2005; Williams et al., 2016).

Wearable devices may be associated with both exercise self-efficacy and positive exercise experiences through multiple pathways. On the one hand, data feedback and goal-achievement prompts provided by wearable devices may correspond to higher perceptions of competence and self-efficacy among users (McAuley et al., 2011; Steel, 2023). On the other hand, gamified features (e.g., badge systems and leaderboards) and social sharing functions may be associated with greater enjoyment and social connectedness during exercise, which may correspond to more positive exercise experiences (Zhao et al., 2015; Sun and Jiang, 2022). Nevertheless, few studies have examined whether exercise self-efficacy and positive experiences jointly explain the association between wearable device use and exercise adherence through a sequential mediation model. Therefore, further investigation of these potential mediation pathways is warranted.

Based on the self-efficacy theory (Bandura, 1997), emotion regulation theory (Gross, 1998), and the Technology Acceptance Model (Davis, 1989; Venkatesh and Bala, 2008), a theoretical framework was constructed in this paper that chains exercise self-efficacy and positive experiences as mediators. Specifically, this study attempts to answer the following three research questions: (1) Is there a statistical association between wearable device use and exercise adherence? (2) Do exercise self-efficacy and positive experiences independently mediate this association? (3) Is a statistically supported sequential mediation pathway involving cognitive and emotional factors present? Because the present study uses a cross-sectional design in which all variables were measured at the same time point, these pathways are specified as theoretically specified statistical pathways rather than as empirically established temporal mechanisms. The results may offer some psychological explanations for long-term exercise behaviour and provide a reference for future studies in technology-assisted exercise promotion.

### 1.1 Wearable device use and exercise adherence

Research based on the Technology Acceptance Model (TAM) suggests that sustained information system use is closely related to perceived usefulness and perceived ease of use (Davis, 1989; Venkatesh and Bala, 2008). In recent years, TAM has been extended to health information technology contexts, with previous studies indicating that continued use intention toward wearable health devices is positively associated with exercise frequency and exercise adherence (Brickwood et al., 2019; Kim and Shin, 2015). Specifically, device functions including activity tracking (e.g., step count, heart rate, and sleep quality monitoring), goal setting, and progress feedback are associated with stronger intentions to participate in physical activity (Warburton et al., 2006; Harkin et al., 2016). Although a randomized controlled trial by Jakicic et al. (2016) found that increased device use alone did not necessarily improve weight-loss outcomes, subsequent analyses suggested that users exposed to higher-functionality features (e.g., data tracking, goal management, and social sharing) reported higher levels of exercise adherence compared with those using lower-functionality features. Therefore, this study proposes:

*H1*: Wearable device use is positively associated with exercise adherence.

Self-efficacy refers to an individual’s belief in their ability to perform specific tasks or behaviours (Bandura, 1997), and its development is primarily influenced by four sources: mastery experiences, vicarious experiences, verbal persuasion, and physiological states (Bandura, 1977; Luszczynska and Schwarzer, 2005). Through functions such as real-time data feedback (e.g., prompts indicating daily step completion), wearable devices may be associated with users’ perceptions of competence and mastery, thereby corresponding to stronger exercise self-efficacy beliefs (McAuley et al., 2011; Steel, 2023). A longitudinal study by Steel (2023) reported that wearable technology use was positively associated with physical activity and self-determined motivation over time, and a meta-analysis of consumer-based activity trackers reported small-to-moderate increases in physical activity participation (Brickwood et al., 2019). Therefore:

*H2*: Wearable device use is positively associated with exercise self-efficacy.

### 1.2 Wearable device use and positive exercise experience

Positive exercise experience represents an important construct in exercise affect research and includes feelings of pleasure, vitality, satisfaction, and flow experienced during exercise (Ekkekakis et al., 2011; Lonsdale et al., 2009). Through gamification features (e.g., challenges, rewards, and badges) and social interaction functions, wearable devices may be associated with greater enjoyment and more positive emotional experiences during exercise (Zhao et al., 2015; Sun and Jiang, 2022). For example, gamified activity-tracking systems that embed challenge, reward, and leaderboard elements have been associated with higher engagement and enjoyment during exercise (Zhao et al., 2015), and music-based interventions are consistently associated with more pleasant affective responses and reduced perceived exertion (Karageorghis and Priest, 2012). Therefore:

*H3*: Wearable device use is positively associated with positive experiences.

### 1.3 Exercise self-efficacy and exercise adherence

The relationship between self-efficacy and exercise adherence represents one of the most extensively studied topics in exercise psychology (McAuley, 1993; Mullan and Markland, 1997). Meta-analytic evidence indicates a moderate association between exercise self-efficacy and adherence-related outcomes, with a weighted average correlation coefficient of approximately *r* = 0.40 (Moritz et al., 2000). This association has also been observed consistently in longitudinal research examining sustained exercise behaviour (McAuley et al., 2011; Teixeira et al., 2012). From a theoretical perspective, individuals with higher self-efficacy are more likely to adopt problem-focused coping strategies when encountering exercise barriers, such as fatigue or limited time, rather than disengaging from planned exercise activities (Bandura, 1997; Luszczynska and Schwarzer, 2005). Therefore:

*H4*: Exercise self-efficacy is positively associated with exercise adherence.

### 1.4 Positive experiences and exercise adherence

Both emotion regulation theory (Gross, 1998) and affective forecasting theory (Wilson and Gilbert, 2003) suggest that positive emotional experiences are related to individuals’ expectations and evaluations of future behaviours. In exercise contexts, positive experiences may strengthen the perceived value of exercise through affective learning processes, thereby being associated with greater willingness to maintain exercise participation (Ekkekakis, 2017. Previous studies have reported that immediate pleasure experienced during exercise is positively associated with exercise adherence over time (Rhodes and Kates, 2015; Kwan and Bryan, 2010). Therefore:

*H5*: Positive experiences are positively associated with exercise adherence.

### 1.5 Exercise self-efficacy and positive experiences

From the perspective of cognitive–emotional interaction, self-efficacy beliefs are related to individuals’ behavioural choices and emotional interpretations during exercise. Individuals with higher self-efficacy may report greater perceptions of control and enjoyment under similar exercise conditions because they tend to interpret physiological responses (e.g., increased heart rate) as signs of engagement and vitality rather than discomfort or fatigue (Ekkekakis, 2017; Lindwall et al., 2014). Longitudinal evidence indicates that exercise self-efficacy is positively associated with continued exercise participation and with more favourable affective evaluations of exercise (McAuley et al., 2011; Ekkekakis et al., 2011). Therefore:

*H6*: Exercise self-efficacy is positively associated with positive experiences.

Integrating the above hypotheses, this study proposes a chain mediation model in which exercise self-efficacy and positive experiences serve as sequential mediators between wearable device use and exercise adherence (MacKinnon, 2008; Taylor et al., 2008). Specifically, wearable device use may be associated with higher levels of exercise self-efficacy through perceived competence and mastery-related experiences (H2). Exercise self-efficacy may further correspond to differences in positive emotional experiences during exercise (H6), which are subsequently associated with exercise adherence (H5). Thus, the proposed model describes a hypothesized statistical pathway of:

$$\begin{array}{l} \text{Wearable device}\;\mathrm{use}\to \text{Exercise self efficacy}\\ \to \text{Positive experiences}\to \text{Exercise adherence} \end{array}$$

representing a potential cognitive–affective–behavioural association pattern. Therefore (Figure 1):

**Figure 1:**
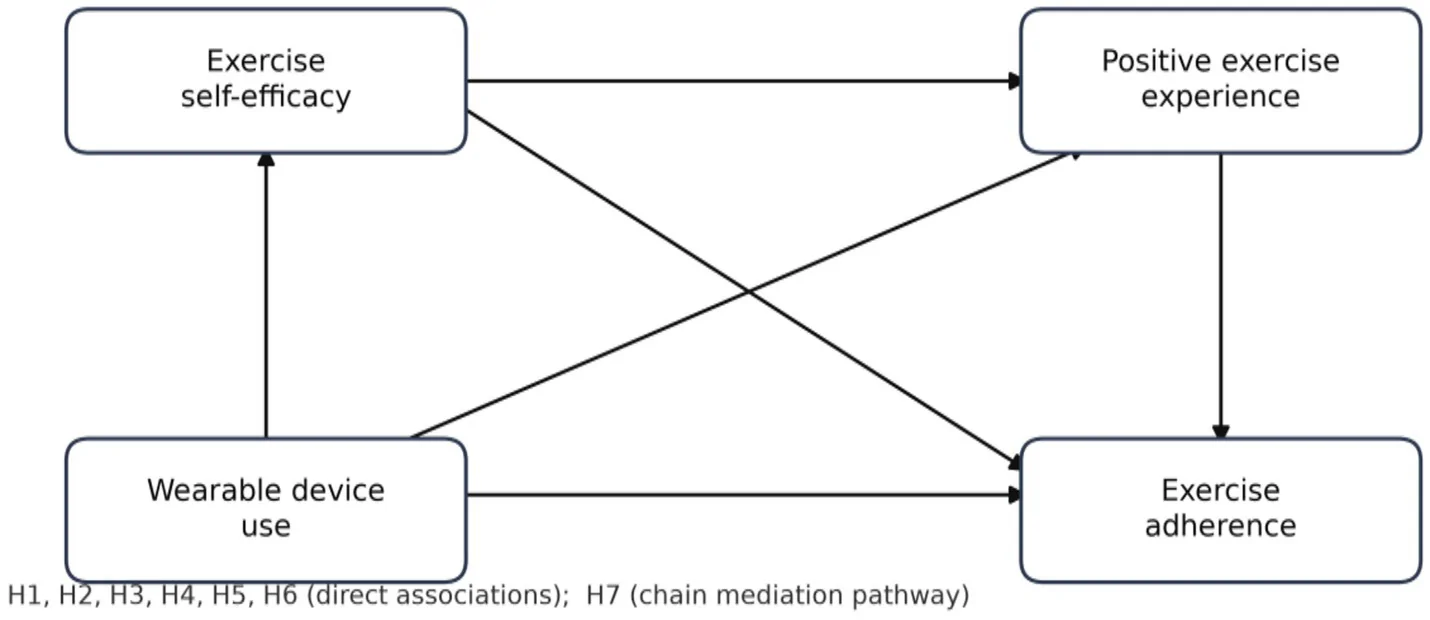
Diagram of the hypothesis model.

*H7*: Exercise self-efficacy and positive experiences form a chain mediation pathway between wearable device use and exercise adherence.

## 2 Methods

### 2.1 Study population

Data were collected between March and May 2026 using a combination of convenience sampling and purposive sampling. Questionnaires were distributed through an online survey platform (Wenjuanxing) and shared with fitness clubs, sports application communities, and college student athlete groups across different provinces and municipalities in China. Recruitment notices containing a QR code and study link were posted in these communities; participation was voluntary and no financial compensation was provided. The inclusion criteria were as follows: (1) having used a smartwatch, fitness tracker, or sports application with data tracking and feedback functions for at least 3 months within the previous 6 months; (2) participating in aerobic exercise or resistance training at least twice per week; and (3) being aged between 18 and 60 years. The questionnaire included two instructed-response attention checks; responses failing either check were excluded. Duplicate responses were identified using platform identifiers, IP address, and response-pattern similarity. A total of 412 questionnaires were collected. After excluding 45 invalid questionnaires (repeated responses, failed attention checks, or completion times shorter than 180 s), 367 valid questionnaires were retained, resulting in an effective response rate of 89.1%. The 180-s cut-off was adopted because the completion-time distribution showed a clear discontinuity at this point and because very short completion times are a well-established indicator of careless responding (Huang et al., 2012; Meade and Craig, 2012). Because the survey platform required a response to every item, no item-level missing data occurred in the retained questionnaires. A Monte Carlo power analysis for the focal structural paths (Wolf et al., 2013) indicated that *N* = 367 provided power of at least 0.95 for detecting the hypothesized coefficients.

The demographic characteristics of the sample were as follows: 195 participants were male (53.1%) and 172 were female (46.9%). Participants ranged in age from 18 to 46 years (*M* = 28.48, SD = 6.76). Regarding educational attainment, 48 participants (13.1%) had a college diploma or lower, 213 (58.0%) had a bachelor’s degree, and 106 (28.9%) had a master’s degree or above. Regarding device usage, 187 participants (51.0%) used smartwatches, 124 (33.8%) used fitness trackers, and 56 (15.3%) used fitness applications independently. In terms of exercise behaviour and technology use, participants exercised on 3.93 days per week on average (SD = 1.18), had been exercising regularly for 4.48 years on average (SD = 2.19), had been using their current device or application for 15.28 months on average (SD = 6.15), and used the technology 5.28 times per day on average (SD = 1.78).

This study was approved by the Ethics Committee of Capital University of Physical Education and Sports (Approval No. 2025–11). All participants provided informed consent before completing the questionnaire. The study was conducted in accordance with the ethical principles outlined in the Declaration of Helsinki.

### 2.2 Measurement tools

#### 2.2.1 Wearable Device Usage Scale

The Wearable Device Usage Scale (WDU) was developed for the present study to assess how frequently respondents used four functional clusters of wearable devices and fitness applications: data-tracking functions (e.g., step, heart-rate, and sleep monitoring), goal-setting and feedback functions (e.g., daily targets and progress prompts), social sharing functions (e.g., sharing workouts and participating in friend challenges), and personalization functions (e.g., customizing displays and personalized recommendations). Item generation was informed by the functional taxonomy of consumer health wearables (Piwek et al., 2016; Ringeval et al., 2020) and by previously validated measures of technology feature use (Kim and Shin, 2015), and the initial item pool was reviewed by five experts in sport and exercise psychology for relevance and clarity. Items with a content validity index below 0.80 were removed, leaving 16 items (four per dimension). The scale was then translated into Chinese following a back-translation procedure and pre-tested in a pilot sample of 32 wearable-device users; items with corrected item–total correlations below 0.40 were revised. All items are rated on a 5-point Likert scale (1 = never, 5 = always). In the current sample, Cronbach’s *α* was 0.904, McDonald’s *ω* was 0.918, corrected item–total correlations ranged from 0.53 to 0.61, and inter-item correlations ranged from 0.26 to 0.54.

#### 2.2.2 Exercise Self-Efficacy Scale

The Exercise Self-Efficacy Scale (ESE) used in this study was adapted from the Chinese version of the Exercise Self-Efficacy Scale originally developed by McAuley (1993) and from Bandura’s (1997) conceptualisation of self-efficacy beliefs. Consistent with the task-specific nature of self-efficacy, 12 items were retained that assess task self-efficacy, barrier self-efficacy, and progress self-efficacy (four items per dimension; e.g., “I am confident that I can complete my planned exercise even when I feel tired”). Items were rated on a 5-point Likert scale (1 = not at all confident, 5 = extremely confident). The scale demonstrated high internal consistency (Cronbach’s *α* = 0.892), and McDonald’s *ω* was 0.910, corrected item–total correlations ranged from 0.57 to 0.62, and inter-item correlations ranged from 0.34 to 0.49.

#### 2.2.3 Positive Exercise Experience Scale

The Positive Exercise Experience Scale (PEE) was developed for the present study to assess affective experiences during exercise across four dimensions: pleasure, vitality, satisfaction, and flow. Item content was adapted from established measures of exercise-related affect, namely the Physical Activity Enjoyment Scale (Kendzierski and DeCarlo, 1991), the Flow State Scale (Jackson and Marsh, 1996), measures of affective response to exercise (Ekkekakis et al., 2011), and measures of exercise-related satisfaction (Lonsdale et al., 2009). Sixty candidate items were generated, translated into Chinese using a back-translation procedure, and reviewed by five experts in exercise psychology; after removing items with content validity indices below 0.80 and semantically redundant items, 16 items (four per dimension) were retained. A pilot study with 32 wearable-device users showed acceptable internal consistency for all four dimensions (*α* ≥ 0.78). Items are rated on a 5-point Likert scale (1 = strongly disagree, 5 = strongly agree). In the current sample, Cronbach’s *α* was 0.913, McDonald’s *ω* was 0.925, corrected item–total correlations ranged from 0.54 to 0.66, and inter-item correlations ranged from 0.26 to 0.57.

#### 2.2.4 Exercise Adherence Scale

The Exercise Adherence Scale (EA) was adapted from the Exercise Adherence Rating Scale (EARS; Newman-Beinart et al., 2017). The original 6-item EARS was expanded to 12 items so that adherence could be assessed across three dimensions: adherence to exercise frequency, adherence to exercise intensity, and adherence to exercise duration (four items per dimension; e.g., “I complete my planned exercise sessions even when my schedule is busy”). Items were translated into Chinese using a back-translation procedure and reviewed by five experts in exercise psychology. Items were rated on a 5-point Likert scale (1 = strongly disagree, 5 = strongly agree). In the current sample, Cronbach’s *α* was 0.887, McDonald’s *ω* was 0.906, corrected item–total correlations ranged from 0.57 to 0.61, and inter-item correlations ranged from 0.32 to 0.48.

### 2.3 Data analysis strategy

SPSS 26.0 was used to conduct descriptive statistics, reliability analyses, and correlation analyses, whereas Mplus 8.6 was used for confirmatory factor analysis (CFA) and structural equation modeling (SEM). Sample size adequacy was evaluated with a Monte Carlo power analysis for the structural model (Wolf et al., 2013). Measurement model. A four-factor CFA with all 56 items as indicators was estimated using maximum likelihood (ML). Because all items used 5-point Likert response formats, we examined whether treating them as continuous was defensible: simulation evidence indicates that with five or more response categories and approximately symmetric distributions, treating Likert items as continuous yields parameter estimates and fit indices that closely approximate those obtained with categorical estimators (Rhemtulla et al., 2012; Li, 2016). The primary CFA therefore treated items as continuous, and *χ*^2^, df, CFI, TLI, RMSEA, and SRMR are reported. A one-factor CFA was also estimated to evaluate common method variance (Podsakoff et al., 2003). Convergent and discriminant validity were evaluated with average variance extracted (AVE), composite reliability (CR), the Fornell–Larcker criterion (Fornell and Larcker, 1981), and the heterotrait–monotrait ratio of correlations (HTMT; Henseler et al., 2015). Reliability was evaluated with Cronbach’s *α*, McDonald’s *ω* (McDonald, 1999; Hayes and Coutts, 2020), corrected item–total correlations, and the range of inter-item correlations. Structural model. The hypothesized model was estimated as a path analysis within the SEM framework using scale-level composite scores, because the research questions concern relations among constructs rather than among individual items; composite scores were computed as the mean of the items of each scale. Because the model contains four observed composite variables and all hypothesized paths among them, the structural model is fully saturated (df = 0). Path coefficients are therefore equivalent to standardized regression coefficients estimated on the correlation matrix, and no global fit indices are available for the structural component; the measurement model reported above provides the relevant fit information. Indirect associations were examined using bias-corrected bootstrap confidence intervals based on 5,000 resamples, and an indirect association was considered statistically significant when the 95% confidence interval did not include zero (Preacher and Hayes, 2008; Taylor et al., 2008; MacKinnon et al., 2013). We additionally report the *R*^2^ of each endogenous variable and the proportion of the total association statistically accounted for by the indirect associations (Preacher and Kelley, 2011). To examine whether the specified ordering of the two mediators was empirically preferable, an alternative model in which positive experiences preceded exercise self-efficacy (WDU → PEE → ESE → EA) was also estimated. In addition, three robustness analyses were conducted: (a) re-estimation of the model after excluding participants who used fitness applications only; (b) comparison of the three device groups; and (c) an examination of dimension-specific associations between the four WDU dimensions and exercise adherence. In addition, Harman’s single-factor test was conducted to assess the potential influence of common-method bias.

## 3 Results

### 3.1 Common method bias test

Harman’s single-factor test was used as a preliminary assessment of potential common method bias. An unrotated exploratory factor analysis was conducted using all 56 measurement items. The results showed that the first principal factor accounted for 26.76% of the total variance, which was below the commonly used threshold of 40% (Podsakoff et al., 2003). In addition, a one-factor CFA (*χ*^2^(1484) = 4440.03, *χ*^2^/df = 2.99, CFI = 0.624, TLI = 0.610, RMSEA = 0.074, SRMR = 0.089) fitted the data substantially worse than the hypothesized four-factor model (*χ*^2^(1478) = 2091.48, *χ*^2^/df = 1.42, CFI = 0.922, TLI = 0.919, RMSEA = 0.034, SRMR = 0.040; Δ*χ*^2^(6) = 2348.55, *p* < 0.001), indicating that a single common factor does not account for the covariance structure of the items. Nevertheless, Harman’s test and the one-factor comparison are relatively insensitive diagnostic procedures, and because all variables were measured with self-report scales at a single time point, common method variance may still have inflated the observed associations. This possibility is explicitly acknowledged in the Limitations section.

### 3.2 Descriptive statistics and correlation analysis

Table 1 presents the descriptive statistics and Pearson correlation coefficients for the main variables. The absolute values of skewness and kurtosis for all variables were below 1.0, suggesting acceptable distributional properties of the data (Kline, 2016). All correlations were statistically significant (*p* < 0.001) (Table 2).

**Table 1:** Descriptive statistics and correlation matrix for each variable (*N* = 367).

Variable	*M*	SD	Skewness	Kurtosis	1	2	3	4
1. WDU	3.399	0.621	−0.181	−0.102	1			
2. ESE	3.572	0.645	−0.191	−0.384	0.476***	1		
3. PEE	3.710	0.619	−0.324	−0.195	0.429***	0.573***	1	
4. EA	3.367	0.641	−0.192	−0.095	0.430***	0.497***	0.492***	1

***indicates a statistically significant difference at *p* < 0.0001.

**Table 2:** Structural equation model path estimates.

Hypothesis	Path	*B*	*β*	SE	*t*	*p*	Result
H1	WDU → EA	0.204	0.197	0.051	3.974	<0.001	Supported
H2	WDU → ESE	0.494	0.476	0.048	10.338	<0.001	Supported
H3	WDU → PEE	0.201	0.202	0.048	4.229	<0.001	Supported
H4	ESE → EA	0.251	0.252	0.054	4.610	<0.001	Supported
H5	PEE → EA	0.272	0.263	0.055	4.931	<0.001	Supported
H6	ESE → PEE	0.458	0.477	0.046	10.009	<0.001	Supported
–	WDU → EA (Total association)	0.444	0.430	0.049	9.106	<0.001	–
–	*R*^2^ (ESE/PEE/EA)	–	0.227/0.360/0.340	–	–	–	–

### 3.3 Confirmatory factor analysis

A confirmatory factor analysis (CFA) with four correlated factors and all 56 items as indicators was estimated. The four-factor model demonstrated acceptable fit: *χ*^2^(1478) = 2091.48, *χ*^2^/df = 1.42 (< 3.00), CFI = 0.922 (> 0.90), TLI = 0.919 (> 0.90), RMSEA = 0.034 (< 0.08), and SRMR = 0.040 (< 0.08) (Hu and Bentler, 1999). All standardized factor loadings were statistically significant (*p* < 0.001) and ranged from 0.56 to 0.69. The four-factor model fitted the data substantially better than a one-factor model (*χ*^2^(1484) = 4440.03, *χ*^2^/df = 2.99, CFI = 0.624, RMSEA = 0.074), supporting the discriminant structure of the four constructs. Because each construct is multidimensional, convergent and discriminant validity were evaluated at the dimension level. Standardized loadings of the 14 dimensions on their respective constructs ranged from 0.72 to 0.82. The average variance extracted (AVE) ranged from 0.55 to 0.66 (> 0.50), and composite reliability (CR) ranged from 0.83 to 0.85 (> 0.70), indicating satisfactory convergent validity. Discriminant validity was further supported by the Fornell–Larcker criterion: the square root of the AVE ranged from 0.74 to 0.81 and exceeded all inter-construct correlations, which ranged from 0.43 to 0.57 (Fornell and Larcker, 1981). In addition, all heterotrait–monotrait ratios (HTMT) ranged from 0.47 to 0.64, below the recommended threshold of 0.85 (Henseler et al., 2015). Reliability coefficients are reported in Table 3.

**Table 3:** Reliability coefficients and item-level diagnostics for all scales and dimensions.

Subscale	*k*	Cronbach’s *α*	McDonald’s *ω*	CITC range	Inter-item *r* range	*M* (SD)
Scale level						
WDU (total)	16	0.904	0.918	0.53–0.61	0.26–0.54	3.399 (0.621)
ESE (total)	12	0.892	0.910	0.57–0.62	0.34–0.49	3.572 (0.645)
PEE (total)	16	0.913	0.925	0.54–0.66	0.26–0.57	3.710 (0.619)
EA (total)	12	0.887	0.906	0.57–0.61	0.32–0.48	3.367 (0.641)
Dimension level						
Data tracking (DT)	4	0.792	0.865	0.57–0.63	0.45–0.52	3.552 (0.738)
Goal setting/feedback (GF)	4	0.794	0.867	0.59–0.63	0.45–0.53	3.448 (0.766)
Social sharing (SS)	4	0.800	0.870	0.58–0.63	0.47–0.53	3.223 (0.779)
Personalization (PZ)	4	0.795	0.867	0.58–0.64	0.43–0.54	3.374 (0.772)
Task self-efficacy (TS)	4	0.778	0.857	0.57–0.59	0.45–0.48	3.681 (0.730)
Barrier self-efficacy (BS)	4	0.781	0.859	0.57–0.60	0.46–0.49	3.431 (0.765)
Progress self-efficacy (PS)	4	0.766	0.851	0.55–0.59	0.43–0.47	3.604 (0.716)
Pleasure (PL)	4	0.816	0.879	0.62–0.66	0.49–0.55	3.836 (0.745)
Vitality (VT)	4	0.805	0.873	0.61–0.64	0.48–0.54	3.716 (0.746)
Satisfaction (SF)	4	0.821	0.882	0.63–0.67	0.51–0.57	3.660 (0.760)
Flow (FL)	4	0.817	0.880	0.63–0.66	0.50–0.55	3.629 (0.764)
Frequency adherence (EF)	4	0.760	0.848	0.54–0.59	0.42–0.47	3.460 (0.724)
Intensity adherence (EI)	4	0.766	0.851	0.56–0.58	0.44–0.48	3.238 (0.740)
Duration adherence (ED)	4	0.769	0.852	0.56–0.58	0.44–0.48	3.403 (0.741)

k, number of items; CITC, corrected item–total correlation. Item-level standardized factor loadings ranged from 0.56 to 0.69; dimension-level loadings ranged from 0.72 to 0.82. Reliability was recomputed for the present sample.

### 3.4 Structural equation model path analysis

The structural equation model was estimated using the maximum likelihood (ML) method. Because the model contains four observed composite variables and all hypothesized paths among them, the structural model is fully saturated (df = 0); fit indices are therefore not available for the structural component, and the measurement model reported above provides the relevant fit information. Figure 2 presents the estimated structural model with standardized path coefficients. Wearable device use was significantly associated with exercise adherence (*β* = 0.197, *p* < 0.001), supporting H1. Wearable device use was also positively associated with exercise self-efficacy (*β* = 0.476, *p* < 0.001) and positive experiences (*β* = 0.202, *p* < 0.001), supporting H2 and H3. Furthermore, exercise self-efficacy (*β* = 0.252, *p* < 0.001) and positive experiences (*β* = 0.263, *p* < 0.001) were positively associated with exercise adherence, supporting H4 and H5. Exercise self-efficacy was significantly associated with positive experiences (*β* = 0.477, *p* < 0.001), supporting H6. The total association between wearable device use and exercise adherence was significant (*β* = 0.430, *p* < 0.001). The model explained 22.7% of the variance in exercise self-efficacy, 36.0% of the variance in positive experiences, and 34.0% of the variance in exercise adherence.

**Figure 2:**
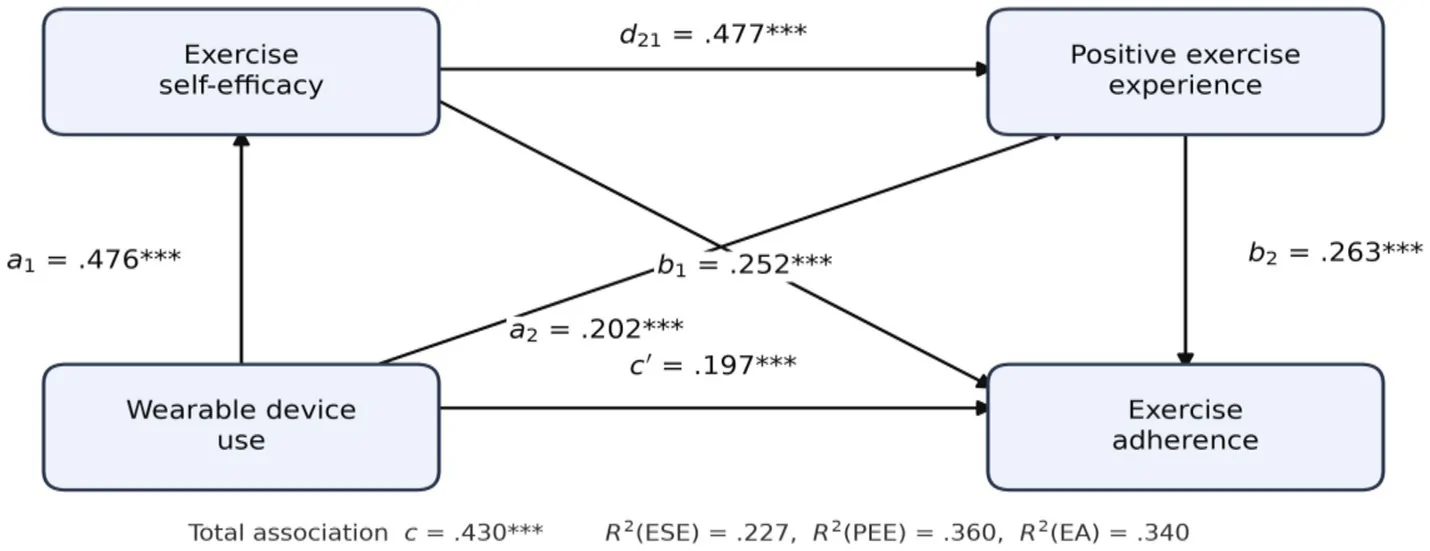
Model validation plot (including standardized path coefficients). ***indicates a statistically significant difference at *p* < 0.0001.

### 3.5 Bootstrap mediation effect test

As shown in Table 4, the 95% confidence intervals of all three indirect pathways did not include zero, indicating statistically significant indirect associations. The indirect association for Path 1 (WDU → ESE → EA) was *β* = 0.119, 95% CI [0.068, 0.176], supporting H2 and H4. The indirect association for Path 2 (WDU → PEE → EA) was *β* = 0.053, 95% CI [0.024, 0.091], supporting H3 and H5. The chained indirect association (WDU → ESE → PEE → EA) was also significant (*β* = 0.059, 95% CI [0.035, 0.086]), supporting H7.

**Table 4:** Bootstrap analysis of indirect associations.

Mediation path	Indirect association (*β*)	SE	Lower 95% CI	Upper 95% CI	Statistical significance
Path 1: WDU → ESE → EA (H2 × H4)	0.119	0.028	0.068	0.176	Significant
Path 2: WDU → PEE → EA (H3 × H5)	0.053	0.017	0.024	0.091	Significant
Path 3: WDU → ESE → PEE → EA (H7 chain)	0.059	0.013	0.035	0.086	Significant
Total indirect association	0.232	0.031	0.176	0.296	Significant
Direct association: WDU → EA	0.197	0.052	0.094	0.298	Significant
Total association: WDU → EA	0.430	0.052	0.326	0.531	Significant

The direct association between wearable device use and exercise adherence remained significant (*β* = 0.197, 95% CI [0.094, 0.298]), suggesting that the relationship between wearable device use and exercise adherence may involve both direct and indirect statistical pathways. The total indirect association was *β* = 0.232, 95% CI [0.176, 0.296], which statistically accounted for 54.0% of the total association (Preacher and Kelley, 2011). This indicates that exercise self-efficacy and positive experiences may represent important explanatory factors in the association between wearable device use and exercise adherence.

Comparison With an Alternative Model. To examine whether the specified ordering of the two mediators was empirically preferable, an alternative model in which positive experiences preceded exercise self-efficacy (WDU → PEE → ESE → EA) was estimated. All paths were statistically significant (WDU → PEE: *β* = 0.429; PEE → ESE: *β* = 0.452; WDU → ESE: *β* = 0.282; ESE → EA: *β* = 0.252; PEE → EA: *β* = 0.263; WDU → EA: *β* = 0.197). However, the chained indirect association in this alternative model was smaller (*β* = 0.049) than in the hypothesized model (*β* = 0.059), and the total indirect association was also smaller (0.162 vs. 0.232). Because cross-sectional data cannot establish temporal ordering, these results indicate that the hypothesized ordering is statistically consistent with the data but do not demonstrate that exercise self-efficacy temporally precedes positive experiences.

Robustness Analyses. Three additional analyses examined the robustness of the main findings. First, because 56 participants (15.3%) used fitness applications only and are therefore not wearable-device users in the conventional sense, the structural model was re-estimated after excluding these participants (*n* = 311). The pattern of associations was unchanged: WDU → ESE *β* = 0.429, WDU → PEE *β* = 0.183, ESE → PEE *β* = 0.464, ESE → EA *β* = 0.240, PEE → EA *β* = 0.298; the direct association was smaller (*β* = 0.138, SE = 0.054, *p* < 0.05) and zero-order correlation between wearable device use and exercise adherence decreased to 0.354, while all indirect associations remained statistically significant. Second, one-way ANOVAs indicated that the three device groups differed significantly in wearable device use (*F*(2, 364) = 97.12, *p* < 0.001), exercise self-efficacy (*F* = 15.21, *p* < 0.001), positive experiences (*F* = 12.70, *p* < 0.001), and exercise adherence (*F* = 16.71, *p* < 0.001), with application-only users scoring lower on all four constructs. Third, the four WDU dimensions were entered simultaneously as predictors of exercise adherence while controlling for exercise self-efficacy and positive experiences; none of the four dimensions was uniquely associated with adherence (βs = 0.043 to 0.077, all *p* > 0.05), whereas exercise self-efficacy (*β* = 0.253, *p* < 0.001) and positive experiences (*β* = 0.263, *p* < 0.001) remained significant. This pattern is consistent with the four dimensions operating jointly rather than through dimension-specific pathways.

## 4 Discussion

### 4.1 The direct association of wearable device use and exercise adherence

The results of this study indicate that wearable device use was positively associated with exercise adherence (*β* = 0.197, *p* < 0.001), supporting Hypothesis H1 and showing consistency with previous research (Brickwood et al., 2019; Kim and Shin, 2015). However, the direct association was smaller than the total association (*β* = 0.430), and the indirect associations statistically accounted for 54.0% of the total association. This pattern suggests that the association between wearable device use and exercise adherence may not reflect a direct behavioural link alone but may also involve cognitive and affective pathways. Because the design was cross-sectional, this pattern should be interpreted as a set of associations consistent with the proposed model rather than as evidence of a causal mechanism.

This pattern may help to interpret the mixed findings reported by Jakicic et al. (2016), who found that simply providing wearable devices within a weight-loss intervention did not improve outcomes. The present results suggest that the association between wearable device use and exercise adherence may be partly attributable to psychological processes such as exercise self-efficacy and positive affective experiences rather than to device provision alone, although alternative explanations (for example, that individuals who already exercise more are more likely to adopt wearable devices) cannot be ruled out with cross-sectional data. Taken together, these observations suggest that designers of exercise technology may wish to consider how device functions might support users’ cognitive beliefs and affective experiences; however, because the present study did not experimentally manipulate any device function, these suggestions should be regarded as tentative design considerations rather than evidence-based intervention recommendations (Verplanken and Wood, 2006).

Furthermore, the significant direct association between wearable device use and exercise adherence suggests that factors beyond the examined psychological mediators may also contribute to this association. These potential pathways may include behavioural processes such as habit formation and situational cues (Verplanken and Wood, 2006; Wood and Rünger, 2016), which were not examined in the current study and warrant further investigation in future research.

### 4.2 The mediating role of exercise self-efficacy

The indirect association of Path 1 (WDU → ESE → EA) was the largest among the three indirect pathways. This finding provides support for Hypotheses H2 and H4 and is consistent with the theoretical assumptions of self-efficacy theory (Bandura, 1997).

Exercise self-efficacy was positively associated with exercise adherence, which is consistent with the theoretical assumptions of the Health Action Process Approach (HAPA; Schwarzer, 2008; Schwarzer et al., 2011). According to HAPA, maintenance self-efficacy refers to individuals’ confidence in maintaining planned behaviours despite potential barriers, such as injury or schedule disruptions, and represents an important factor associated with long-term behavioural maintenance. Individuals with higher self-efficacy may be more likely to adopt cognitive reappraisal strategies (e.g., “I can resume exercise after a temporary interruption”) and problem-focused coping strategies (e.g., preparing alternative exercise plans when facing barriers), rather than discontinuing exercise participation (Luszczynska and Schwarzer, 2005; Teixeira et al., 2012).

The relatively strong association between wearable device use and exercise self-efficacy observed in this study suggests a meaningful relationship between these two variables. This association may be related to the capacity of wearable technologies to provide users with continuous performance information, feedback, and goal-monitoring opportunities. Compared with traditional exercise prescriptions that provide relatively fixed recommendations (e.g., three sessions per week or 30 min per session), wearable devices may offer more individualised feedback experiences, which could correspond to users’ perceptions of competence and progress. This interpretation is consistent with the concept of challenge–competence balance proposed by Csikszentmihalyi (1990), although further longitudinal research is needed to examine these potential processes.

### 4.3 The emotional pathway through positive exercise experiences

The indirect association of Path 2 (WDU → PEE → EA) supported Hypotheses H3 and H5. This finding is consistent with the theoretical assumptions of emotion regulation theory (Gross, 1998) and previous research on exercise-related emotions (Williams et al., 2016; Ekkekakis et al., 2021).

Wearable device use may be associated with more positive emotional experiences during exercise through several potential pathways. Gamification features, including achievement badges, challenges, and leaderboards, may correspond to greater enjoyment and engagement during exercise (Zhao et al., 2015). Social interaction functions, such as friend challenges, workout sharing, and virtual encouragement, may facilitate perceptions of social connectedness and recognition (Sun and Jiang, 2022). In addition, personalized music and audio content may provide richer sensory experiences and help users manage perceptions of exercise fatigue (Karageorghis and Priest, 2012). Collectively, these functions may contribute to more positive interpretations of exercise experiences.

The positive association between positive experiences and exercise adherence can be interpreted from the perspective of affect–behaviour congruency (Ferguson et al., 2011). Positive emotional experiences during exercise may be encoded as favourable affective representations associated with exercise behaviour through implicit memory processes. When individuals evaluate future exercise participation, these affective representations may contribute to more favourable behavioural intentions and willingness to engage in exercise (Woolley and Fishbach, 2016). This perspective provides a possible explanation for individual differences in long-term exercise adherence, although longitudinal studies are needed to examine the temporal relationship between exercise experiences and adherence behaviour.

The association between wearable device use and positive experiences remained significant after exercise self-efficacy was taken into account, although it was weaker than the zero-order correlation between these variables (r = 0.429). This finding suggests that wearable device features related to gamification and social interaction may represent an additional pathway associated with positive exercise experiences beyond self-efficacy-related factors. Therefore, the findings provide preliminary support for a potential dual-pathway perspective involving both functional cognition and emotional experience.

### 4.4 Theoretical implications of the chain mediation pathway

The chain mediation pathway proposed in this study (WDU → ESE → PEE → EA) was statistically supported, providing support for Hypothesis H7. The findings suggest a potential cognitive–affective–behavioural association pattern in which wearable device use is associated with exercise self-efficacy, which is further associated with positive exercise experiences and, subsequently, exercise adherence. Specifically, wearable device use may correspond to greater perceptions of competence through feedback and mastery-related experiences, while higher exercise self-efficacy may be related to more positive interpretations of exercise experiences. These positive experiences are further associated with higher levels of exercise adherence. It should be emphasised that this sequence was specified *a priori* on theoretical grounds; the data support its statistical plausibility but cannot establish the temporal ordering of the two mediators.

This statistical pathway provides empirical support for the relevance of cognitive–emotional interactions in understanding exercise-related behaviours and is consistent with the core assumptions of cognitive appraisal theory (Lazarus and Folkman, 1984). According to this perspective, emotional responses are shaped not only by objective stimuli but also by individuals’ cognitive evaluations of those stimuli. In the context of exercise technology, self-efficacy beliefs may represent an important cognitive factor linking technology-related feedback experiences and emotional evaluations of exercise. The significant association between exercise self-efficacy and positive experiences observed in this study (ESE → PEE, *β* = 0.476) is consistent with this theoretical interpretation.

Based on the extended Technology Acceptance Model (TAM), this chain mediation model further investigates how behaviour related to technology is linked with continuous practice of health behaviour. Wearable device use can be regarded as technology-related behavioural engagement, and exercise self-efficacy and positive experiences are cognitive and affective factors that may motivate people to exercise voluntarily. It should be noted, however, that core TAM constructs such as perceived usefulness and perceived ease of use were not directly measured in this study; the present findings therefore cannot be interpreted as an extension or a test of TAM itself. Rather, they describe potential psychological pathways that may connect wearable technology use with exercise adherence in a health behaviour context (Venkatesh and Bala, 2008; Kim and Shin, 2015).

Because the model specifies a chain of mediation, multiple psychological factors may operate jointly in the association between wearable device use and exercise adherence. Future technology-assisted exercise interventions might therefore consider combining cognitive-oriented elements (goals and feedback) with affective-oriented elements (gamification and social interaction); however, because the present study is cross-sectional and did not manipulate these features, such suggestions remain tentative. Longitudinal or experimental research would be needed to determine whether integrated approaches can effectively support long-term exercise adherence.

## 5 Research limitations and future directions

This study provides preliminary evidence for the association between wearable device use and exercise adherence, but several limitations should be acknowledged.

First, the cross-sectional design limits inferences about causality. Only one survey was conducted; although SEM can represent the structure of associations among variables, it cannot rule out the influence of unobserved third variables or establish the temporal ordering of the proposed pathways. Cross-sectional estimates of longitudinal mediation are known to be biased (Maxwell and Cole, 2007), and the present results should therefore be read as associations consistent with the hypothesized model rather than as evidence of causal processes. In future research, multi-wave designs with several observation points could be combined with techniques such as random-intercept cross-lagged panel models (Hamaker et al., 2015) or random-effects models to determine whether these associations are stable over time. In addition, we tested a theoretically plausible alternative ordering of the mediators; because the fit of competing orderings cannot be distinguished with cross-sectional data, future longitudinal work is required to compare them directly.

Second, all measures were self-reported and may be subject to social desirability and recall bias. Although Harman’s single-factor test and the comparison of one-factor and four-factor confirmatory models did not indicate that a single common factor accounted for the covariance structure of the items, these procedures are relatively insensitive, and common method variance may still have inflated the observed associations. In addition, exercise adherence was assessed exclusively by self-report; individuals with favourable attitudes toward wearable devices may systematically overestimate their exercise behaviour (Sallis and Saelens, 2000). Future research should combine objective behavioural indicators—such as device log data and accelerometer measurements—with self-report scales to create a multi-method assessment system (Sallis and Saelens, 2000).

Third, the sample was not representative of the general adult population. Participants were recruited through convenience and purposive sampling from fitness clubs, sports-application communities, and student athlete groups, and all were required to have used the technology for at least 3 months and to exercise at least twice per week. The sample therefore represents physically active and technology-engaged individuals rather than the general adult population. Selection bias and range restriction are likely: inactive individuals, technology dropouts, and individuals who discontinued exercise are under-represented or were excluded by the inclusion criteria. Consequently, the findings should be generalised only to physically active users of digital fitness technologies, and future research should recruit more representative samples, including older adults and individuals with limited mobility.

Fourth, this study used wearable device use as an overall construct and did not separate its functional dimensions in the main model. A supplementary analysis in which the four dimensions were entered simultaneously showed that none of the dimensions was uniquely associated with exercise adherence, which is consistent with the dimensions operating jointly; nevertheless, larger samples with sufficient power for dimension-level tests would be needed to examine dimension-specific pathways more conclusively.

Fifth, the internal consistency coefficients were high for the total scales, and although the values reported here (*α* = 0.887–0.913; *ω* = 0.906–0.925) are considerably lower than those originally reported and are accompanied by acceptable item-level diagnostics, high coefficients can also reflect item redundancy or limited construct breadth. Future work should therefore examine the dimensionality and breadth of these scales further, for example through item-response-theory analyses. Sixth, the cultural context of this study may limit its cross-cultural applicability; future research could conduct cross-cultural comparisons to determine whether the presented associations generalise to other cultures.

## 6 Conclusion

This study examined a hypothesized theoretical framework linking wearable device use with exercise adherence through a chain mediation model involving exercise self-efficacy and positive experiences (N = 367). The findings are consistent with, but do not confirm, the proposed directional model. The main findings are summarized as follows:

1. Wearable device use was positively associated with exercise adherence (direct association *β* = 0.197; total association *β* = 0.430), and three statistically significant indirect association pathways were identified.
2. Exercise self-efficacy represented an indirect association pathway between wearable device use and exercise adherence (*β* = 0.119, 95% CI [0.068, 0.176]). Among the three indirect pathways, this pathway showed the largest indirect association magnitude, which is consistent with the relevance of self-efficacy theory in understanding exercise technology-related behaviours.
3. Positive experiences also represented an independent indirect association pathway between wearable device use and exercise adherence (*β* = 0.053, 95% CI [0.024, 0.091]), suggesting that emotional experiences may represent an important pathway linking wearable device use with exercise adherence.
4. Exercise self-efficacy and positive experiences formed a statistically supported chain mediation pathway (*β* = 0.059, 95% CI [0.035, 0.086]). The indirect associations statistically accounted for 54.0% of the total association, highlighting the potential relevance of cognitive–emotional interactions in understanding exercise adherence among wearable device users.

Based on these findings, future wearable technology design may wish to consider integrating both cognitive-oriented functions (e.g., performance feedback and goal-setting systems) and affective-oriented features (e.g., gamification, social interaction, and personalized recommendations) to support sustained exercise adherence; however, because the present study did not manipulate these features, these suggestions should be treated as tentative design considerations. Longitudinal and experimental studies are needed to examine whether such design strategies can effectively improve long-term exercise adherence, and to test the directionality of the associations reported here.

## Data Availability

The raw data supporting the conclusions of this article will be made available by the authors, without undue reservation.
